# The maturation of the P1m component in response to voice from infancy to 3 years of age: A longitudinal study in young children

**DOI:** 10.1002/brb3.1706

**Published:** 2020-06-23

**Authors:** Yuko Yoshimura, Chiaki Hasegawa, Takashi Ikeda, Daisuke N. Saito, Hirotoshi Hiraishi, Tetsuya Takahashi, Hirokazu Kumazaki, Mitsuru Kikuchi

**Affiliations:** ^1^ Institute of Human and Social Sciences Kanazawa University Kanazawa Japan; ^2^ Research Center for Child Mental Development Kanazawa University Kanazawa Japan; ^3^ Institute for Medical Photonics research Hamamatsu University school of medicine Hamamatsu Japan; ^4^ Health Administration Center University of Fukui Fukui Japan; ^5^ Institute of Medical Pharmaceutical and Health Sciences Kanazawa University Ishikawa Japan

**Keywords:** auditory‐evoked field (AEF), magnetoencephalography (MEG), young children

## Abstract

**Introduction:**

In the early development of human infants and toddlers, remarkable changes in brain cortical function for auditory processing have been reported. Knowing the maturational trajectory of auditory cortex responses to human voice in typically developing young children is crucial for identifying voice processing abnormalities in children at risk for neurodevelopmental disorders and language impairment. An early prominent positive component in the cerebral auditory response in newborns has been reported in previous electroencephalography and magnetoencephalography (MEG) studies. However, it is not clear whether this prominent component in infants less than 1 year of age corresponds to the auditory P1m component that has been reported in young children over 2 years of age.

**Methods:**

To test the hypothesis that the early prominent positive component in infants aged 0 years is an immature manifestation of P1m that we previously reported in children over 2 years of age, we performed a longitudinal MEG study that focused on this early component and examined the maturational changes over three years starting from age 0. Five infants participated in this 3‐year longitudinal study.

**Results:**

This research revealed that the early prominent component in infants aged 3 month corresponded to the auditory P1m component in young children over 2 years old, which we had previously reported to be related to language development and/or autism spectrum disorders.

**Conclusion:**

Our data revealed the development of the auditory‐evoked field in the left and right hemispheres from 0‐ to 3‐year‐old children. These results contribute to the elucidation of the development of brain functions in infants.

## Graphical abstract

We performed a longitudinal magnetoencephalography (MEG) study that focused on auditory early component. The maturational changes over three years starting from age 0 were examined. Our result revealed that the early prominent component in infants aged 3 month corresponded to the auditory P1m component in young children over 2 years old, which we had previously reported to be related to language development and/or autism spectrum disorders.

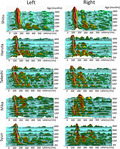

**Abbreviations**
AEF: Auditory‐evoked field
MEG: magnetoencephalography
TD: typically developing

## 1 INTRODUCTION

### 1.1 Maturation in the central auditory system

In infancy and early childhood, the connections between neurons in the brain are highly malleable. During functional development, the myelination of intracortical nerve axons is already in progress at birth and continues for two decades or more (Herschkowitz, 1988). Cortical maturation processes, such as myelination and synaptic formation, are crucial for neurodevelopment and support and enable cognitive and behavioral development. A large number of previous studies have focused on brain maturation using noninvasive electrophysiological methods, such as electroencephalography (EEG), from newborns to children. Since auditory stimulation can be easily applied to young children who are otherwise uncooperative, many previous studies have focused on the maturational trajectory of the brain auditory response. Ponton et al showed age‐related changes in the latency and magnitude of the auditory‐evoked potentials (AEPs) to pure tone stimuli (Ponton, Eggermont, Khosla, Kwong, & Don, 2002). In addition, Kurtzberg et al recorded AEPs to tones and speech sounds in normal and very low birth weight infants, and they reported that these infants showed immature AEP patterns at term (Kurtzberg, 1982; Kurtzberg, Hilpert, Kreuzer, & Vaughan, 1984). Jing and Benasich (2006) also investigated the event‐related potentials (ERPs) in five healthy infants monthly between the ages of 3 and 24 months and reported that latencies of ERPs to tones decreased with age.

### 1.2 Language acquisition and maturation in the central auditory system

Intriguingly, these neurophysiological methods have allowed researchers to examine complex cognitive processes such as language and communication development (Friederici, 2005). In typically developing children without any language disabilities, native‐language phonetic perception is thought to represent a critical step in initial language learning and promote language growth (Kuhl, 2010; Kuhl et al., 2006; Tsao, Liu, & Kuhl, 2004). Using magnetoencephalography (MEG), brain responses to human voices have been studied as a physiological indicator of language acquisition (Imada et al., 2006; Kuhl, 2010; Kuhl, Ramirez, Bosseler, Lin, & Imada, 2014; Yoshimura et al., 2012, 2014). Therefore, in children with language disorders, the majority of previous studies have also focused on brain responses to human voices. Some previous studies focused on the responses to syllables(Breier et al., 2003; Heim, Eulitz, & Elbert, 2003; Heim et al., 2000; Paul, Bott, Heim, Eulitz, & Elbert, 2006; Paul, Bott, Heim, Wienbruch, & Elbert, 2006; Pihko et al., 2007, 2008), while others focused on responses to word stimuli (Helenius et al., 2014; Mody, Wehner, & Ahlfors, 2008; Wehner, Ahlfors, & Mody, 2007). With regard to the brain responses to syllabic auditory stimuli, Pihko et al. (2008) demonstrated a reduced magnitude in the early prominent component (i.e., P1m) in both hemispheres in 5‐ to 7‐year‐old children with specific language impairment (SLI) (Pihko et al., 2008) and, intriguingly, they also demonstrated that phonological intervention enhances P1m magnitude in both hemispheres in 6‐ to 7‐year‐old children with SLI (Pihko et al., 2007). Based on the results of these previous studies, we have focused on the development of auditory processing in early childhood by measuring auditory‐evoked magnetic fields (AEFs) using child‐customized MEG. We reported the traits of the auditory response in typically developing preschool children and children with autism spectrum disorder (ASD) in relation to language acquisition (Yoshimura et al., 2012, 2013, 2014, 2016). The uniqueness of our research is that we employ the vocalized syllable/ne/ as an auditory stimulus. In Japanese,/ne/ is often used in mother–child conversations and expresses the speaker's request for joint attention with the listener (Kajikawa, Amano, & Kondo, 2004; Squires, 2009). Given that the development of joint attention is linked to language development (Tomasello, & Haberl, 2003), we thought that the brain response to a human vocalization of the syllable/ne/ would be a possible physiological indicator of language acquisition. Using this human voice stimulus, we have reported that a higher intensity in the early prominent component (i.e., P1m) is related to a higher language conceptual ability in typically developing children (Yoshimura et al., 2012, 2013, 2014, 2016).

### 1.3 Early prominent component in the cerebral auditory‐evoked response

Previous AEP (EEG) studies have reported the detection of the early positive component in central and frontal electrodes immediately after auditory stimulation in young children, and this early component has often been called P1 (C. W. Ponton, Eggermont, Kwong, & Don, 2000; Sharma, Kraus, McGee, & Nicol, 1997). Previous EEG and MEG studies reported that this early positive component appears approximately 100 ms after auditory stimulation in children over 3 years old (Gilley, Sharma, Dorman, & Martin, 2005; Oram Cardy, Ferrari, Flagg, Roberts, & Roberts, 2004; Ponton et al., 2002) and grows larger during childhood and eventually decreases in adulthood (Ponton et al., 2002). In previous MEG studies, various names have been given for this early prominent component, for example, M50 (Oram Cardy et al., 2004; Oram Cardy, Flagg, Roberts, Brian, & Roberts, 2005; Oram Cardy, Flagg, Roberts, & Roberts, 2008; Roberts et al., 2010), P1m (Pihko et al., 2007, 2008), or P50m (Menning, Ackermann, Hertrich, & Mathiak, 2005; Onitsuka, Ninomiya, Sato, Yamamoto, & Tashiro, 2000; Tavabi, Obleser, Dobel, & Pantev, 2007). We have labeled this early, most prominent component P1m and have reported cross‐sectional (Yoshimura et al., 2013, 2016) and longitudinal studies (Yoshimura et al., 2014) on the maturational process of the magnitude of the current source for children aged 2 to 10 years.

### 1.4 Significance of a longitudinal study on the early prominent component of AEFs in 0‐ to 3‐year‐old infants.

Understanding the typical developmental patterns of the maturation of the AEF/AEP evoked by speech sounds from infants aged 0 years may aid in the development of objective early diagnosis techniques for abnormal central auditory maturation related to speech, language, communication, and learning impairments. In our previous reports, since our subjects were children aged two years or older, the developmental trajectory of the P1m component evoked by voice stimuli before 2 years of age had not been clarified. However, a number of studies have reported that the positive component is first obvious in the auditory response (auditory‐evoked potential (AEP), AEF) soon after birth (Edgar et al., 2015; Holst et al., 2005; Kushnerenko, Ceponiene, Balan, Fellman, & Naatanen, 2002; Lippe, Martinez‐Montes, Arcand, & Lassonde, 2009; Lutter, Maier, & Wakai, 2006; Ortiz‐Mantilla & Benasich, 2013; Wunderlich, Cone‐Wesson, & Shepherd, 2006). However, it is still unknown whether these positive components correspond to the speech‐evoked P1m that we previously reported in 2‐ to 10‐year‐old children. To confirm this possibility, a longitudinal study targeting children aged 0–3 years is necessary. Our purpose in this study is to investigate the age‐related changes in voice‐evoked responses in 0‐ to 3‐year‐old infants. We hypothesized that the prominent early positive component in infants would show a decrease in latency with age and correspond to the P1m component that we reported in children aged 2–10 years. To confirm this, we investigated the developmental trajectory of voice‐evoked responses in five typically developing children from 3 to 36 months using child‐customized MEG.

## 2 MATERIALS AND METHODS

### 2.1 Participants

Five (four boys and one girl) healthy children participated in this study. To avoid providing identifying information in this report, the names given to the children here are Shizu, Haruta, Takeshi, Mika, and Syun. Participants were 2 months old at the first measurement. The measurements took place at approximately 1‐month interval. All 5 children were tested until 36 months of age. No child had any developmental issues at 36 months. All participants had normal hearing according to their newborn auditory screening and available medical records. The parents agreed to allow their child to participate in the study and had full knowledge of the experimental nature of the research. Written informed consent was obtained prior to participation in the study. The Ethics Committee of Kanazawa University Hospital approved the methods and procedures, which were performed in accordance with the Declaration of Helsinki.

### 2.2 Magnetoencephalography recordings

MEG data were recorded using a 151‐channel superconducting quantum interference device (SQUID) and a whole‐head coaxial gradiometer MEG system for children (PQ 1151R; Yokogawa/KIT, Kanazawa, Japan) in a magnetically shielded room (Daido Steel) installed at the MEG Center of Ricoh Company, Ltd. The custom child‐sized MEG system facilitates the measurement of brain responses in young children, which would otherwise be difficult using conventional adult‐sized MEG systems. The child‐sized MEG system ensures that the sensors are easily and effectively positioned for the child's brain and that head movements are constrained (Johnson, Crain, Thornton, Tesan, & Reid, 2010). The MEG measurement started after confirmation that the head of the subject was located in the center of the MEG helmet by measuring three or four locations on the surface of the head, which served as fiduciary points relative to specific landmarks (the bilateral mastoid processes, Cz, and 5 cm from Cz to nasion). An experimenter and the child's mother remained in the room to encourage the child, to keep him or her awake, and to prevent movement throughout the MEG recording. Stimuli were presented while the child was in a supine position on the bed and viewed video programs projected onto a screen.

### 2.3 AEF stimuli and procedures

**FIGURE 1:**
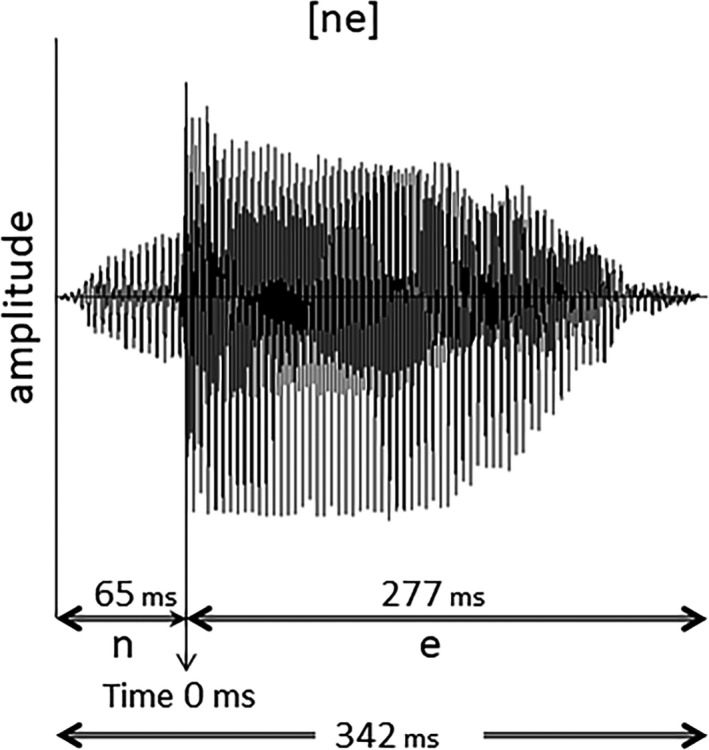
Waveform of the/ne/ speech stimulus. The total duration was 342 ms, with 65 ms for the consonant/n/ and 277 ms for the postconsonantal vowel sound/e/. MEG averaging started at the onset of the/e/ sound

MEG recordings were obtained from all participants during auditory stimulation with the Japanese syllable/ne/ (Yoshimura et al., 2012; Figure 1). We used this syllable because/ne/ is one of the final sentence particles used in Japanese, which conveys prosodic information (Anderson, Hiramoto, & Wong, 2007; Cook, 1990). The syllable/ne/ is often used in Japanese mother–child conversations and expresses a speaker's request for acknowledgement or empathy from the listener (Kajikawa et al., 2004; Squires, 2009). In the present study, we used typical oddball sequences consisting of standard stimuli (456 times, 83%) and deviant stimuli (90 times, 17%). In the standard stimulus,/ne/ was pronounced with a steady pitch contour, whereas in the deviant condition,/ne/ was pronounced with a falling pitch. Eventually, we adopted only the standard stimuli for subsequent equivalent current dipole (ECD) estimations because a sufficient number of periods to calculate ECD remained after artifact rejection in all children. A female native Japanese speaker produced the/ne/ sounds, which were recorded using a condenser microphone (NT1‐A; Rode) and a personal computer. As shown in Figure 1, the duration of the stimulus was 342 ms, and the duration of the consonant/n/ was 65 ms. In this study, the beginning of the vowel sound/e/ was defined as the onset time. The interstimulus interval (ISI) was 818 ms. Each stimulus had an intensity level of approximately 65 dB (A‐weighted) at the head position against a background noise level of 43 dB. Intensity was measured using an integrating sound level meter (LY20; Yokogawa). The stimulus was presented to the participants binaurally through tubes fixed to the dewar. The recording was 12 min long.

### 2.4 AEF acquisition and analysis

The bandpass‐filtered MEG data (0.16–200 Hz) were collected at a sampling rate of 2,000 Hz. The time series from −150 to 1,000 ms relative to the onset of the syllable stimulus and subsequent segments were averaged for each sensor after baseline correction (using the data from −50 to 0 ms). The number of trials after artifact rejection was 373 ± 88 (mean ± *SD*). Segments contaminated with artifacts (eye‐blinks and body movements, typically more than ± 4 pT) were excluded automatically from the analysis. In addition, we visually identified artifacts that resulted from body and face movements using video recorded during the measurements and excluded epochs that contained such artifacts from the analysis. A single ECD model was used to estimate current sources in the activated cerebral cortex using >49 sensors for each hemisphere (left and right). MegLaboratory 160 software (Yokogawa/KIT) was used to estimate the localization of the current sources. Although we could not take into account how the individual head shape would influence the accuracy of the dipole estimation, the ECD could still be calculated without magnetic resonance imaging anatomical data. A sphere, acting as a spherical model of the volume conductor, was fitted to the center of the helmet after confirmation that the head of each subject was located in the center of the MEG helmet by measuring three or four locations on the surface of the head, which served as fiduciary points relative to specific landmarks (the bilateral mastoid processes, Cz, and 5 cm from Cz to nasion). To identify P1m, we first accepted the estimated ECDs if (a) the goodness of fit (GOF) exceeded 80%; (b) the locations of the estimated dipoles using a single ECD model were stabilized within ± 5 mm of each coordinate for at least 6 ms during the P1m response; (c) the dipole amplitudes were ≤ 80 nAm; and (d) the ECDs predominantly had an anterosuperior direction. The latency was defined as the time point when the estimated dipole intensity value reached a maximum and met the above criteria within the time window between 75 and 235 ms.

### 2.5 Statistical analysis

Statistical analyses were conducted using SPSS for Windows statistical software, version 20.0 (IBM). To evaluate the relationships between the dipole intensity (or latency) of the P1m component and age in months, the Jonckheere–Terpstra test was used. The alpha level was set to 0.025 (Hasegawa et al., 2018).

## 3 RESULTS

**FIGURE 2:**
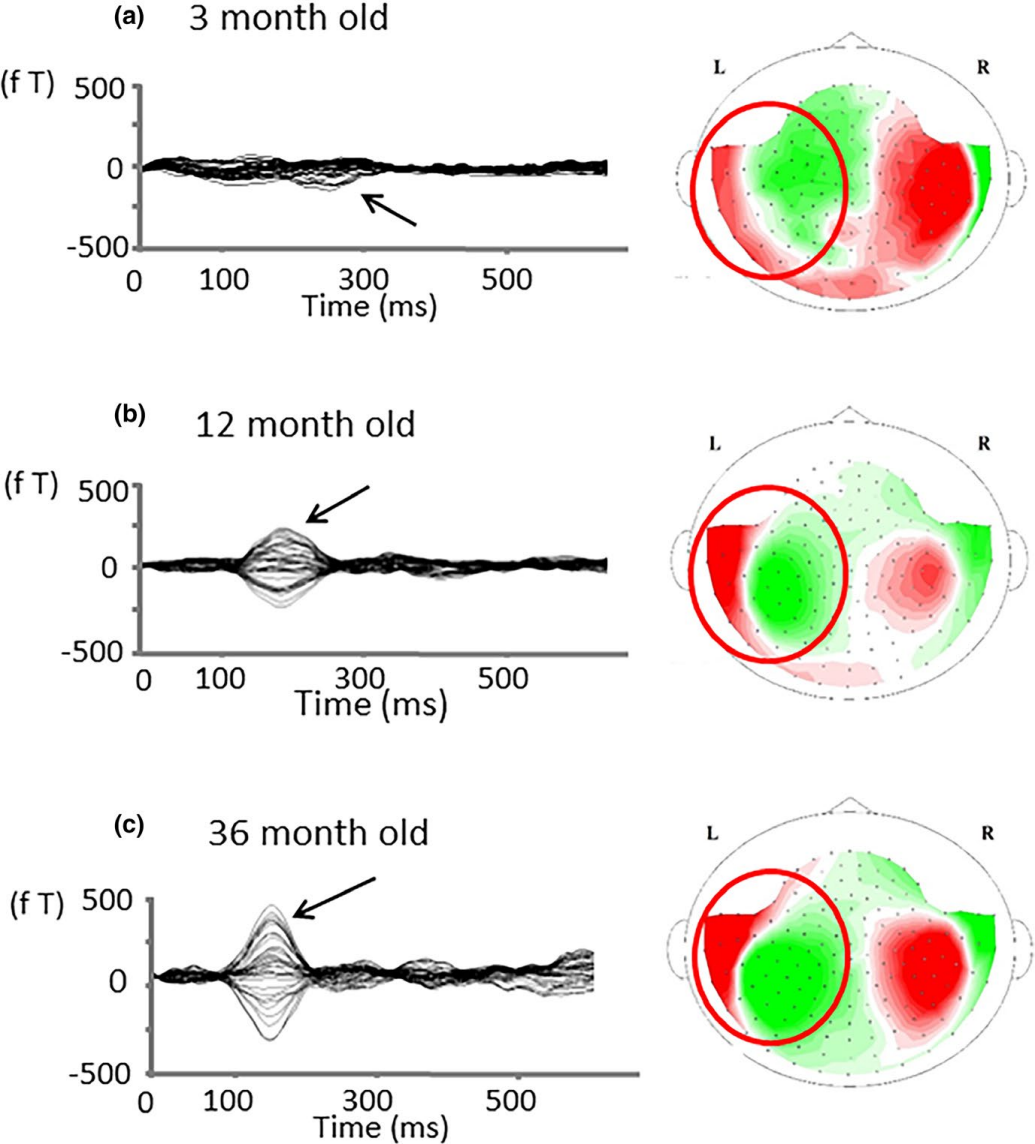
AEF waveform and sensor‐level topography for the early prominent positive component in a child at 3 different ages in months. (left) AEF waveforms and (right) sensor‐level contour maps for the early prominent positive component in a child at 3 different ages in months. Left: AEF waveforms at (a) 3 months, (b) 12 months, and (c) 36 months of age. The arrows indicate the early prominent positive component. The sensors in the red open circles were used the waveform in the left part of the figure

**FIGURE 3:**
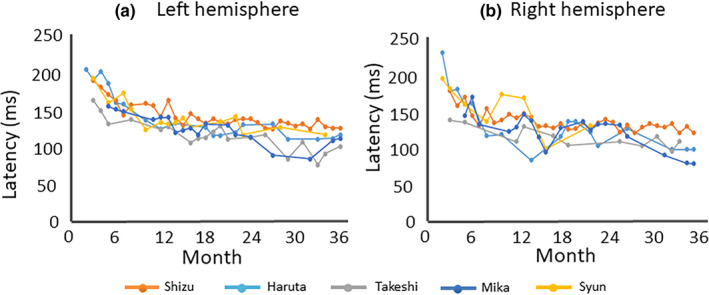
Developmental trajectory of P1m latency. Left hemisphere (a) and right hemisphere (b). In this age range, the P1m latency nearly constantly decreased with age

The number of MEG measurements from each participant and the number of detected early positive prominent components in the left and right hemispheres are shown in Table 1. The number of cases where ECD modeling could be performed is shown in the Table S1. The latency (mean ± *SD*) of P1m for each month of age is shown in Table 2. As shown in Figure 2, different auditory‐evoked waveforms were observed among the different ages in months, as predicted. The Jonckheere–Terpstra test revealed a significant decrease in P1m latency with age in the left hemisphere (TJT = 98.5, *SE* = 39.3, *z* = −5.514, *p* < .001) and right hemisphere (TJT = 100.0, *SE* = 33.3, *z* = −4.554, *p* < .001; Figure 3).

**TABLE 1:** The number of MEG measurements for each participant and the number of detectable early positive prominent components in the left and right hemispheres

Name	Age in months (MEG recorded)	Number of detectable early prominent components (number of MEG measurements)	
		Left	Right
Shizu	3,4,5,6,7,8,9,10,11,12,13,14,15,16,17,18,19,20,21,22,23,24,25,26,27,28,29,30,31,32,33,34,35,36	33 (34)	34 (34)
Haruta	2, 3, 4, 5, 6, 7, 8, 9, 10,11, 12, 14, 17, 18, 19, 20, 22, 23, 27, 29, 33, 35, 36	19 (23)	17 (23)
Takeshi	2, 4, 5, 6, 7, 8, 10, 11, 12, 13, 14, 15, 16, 17, 18, 20, 21, 22, 23, 24, 26, 27, 32, 35, 36	18 (25)	19 (25)
Mika	3, 4, 5, 8, 9, 12, 13, 16, 17, 18, 19, 20, 21, 24, 26, 29, 31, 32, 33, 34, 36	19 (21)	11 (21)
Syun	2, 3, 5, 7, 8, 10, 12, 13, 14, 15, 16, 20, 22,23,28,34	15 (16)	8 (16)

**TABLE 2:** Changes in the latency of P1m in the left and right hemispheres

Age in months	*N*	Left hemisphere (ms) Mean (*SD*)	*N*	Right hemisphere (ms) Mean (*SD*)
2	1	211	2	214
3	4	189 (14)	4	169 (21)
4	3	182 (27)	2	169 (16)
5	5	165 (20)	5	153 (14)
6	3	163 (6)	3	158 (13)
7	4	160 (13)	2	131 (21)
8	4	153 (10)	3	135 (19)
9	‐	–	1	134
10	3	144 (18)	3	143 (28)
11	2	151 (13)	2	134 (16)
12	5	137 (8)	3	125 (16)
13	4	145 (16)	4	147 (16)
14	4	135 (8)	3	120 (33)
15	3	135 (8)	2	121 (10)
16	4	130 (16)	3	107 (20)
17	3	126 (14)	2	121 (7)
18	4	130 (9)	3	125 (9)
19	3	129 (12)	3	121 (16)
20	4	132 (8)	2	131 (7)
21	3	128 (14)	2	135 (1)
22	4	133 (11)	4	125 (3)
23	3	133 (12)	3	122 (17)
24	3	126 (11)	2	136 (4)
25	1	138 (14)	1	135
26	2	125 (6)	3	120 (11)
27	3	119 (23)	3	124 (8)
28	2	119 (6)	1	120
29	3	113 (24)	2	115 (18)
30	1	133	1	133
31	2	123 (17)	2	122 (10)
32	2	108 (28)	2	108 (27)
33	3	112 (31)	3	108 (21)
34	3	116 (19)	2	114 (8)
35	3	119 (9)	3	101 (25)
36	4	116 (10)	3	98 (21)

Abbreviation: *N*, number of subjects.

**FIGURE 4:**
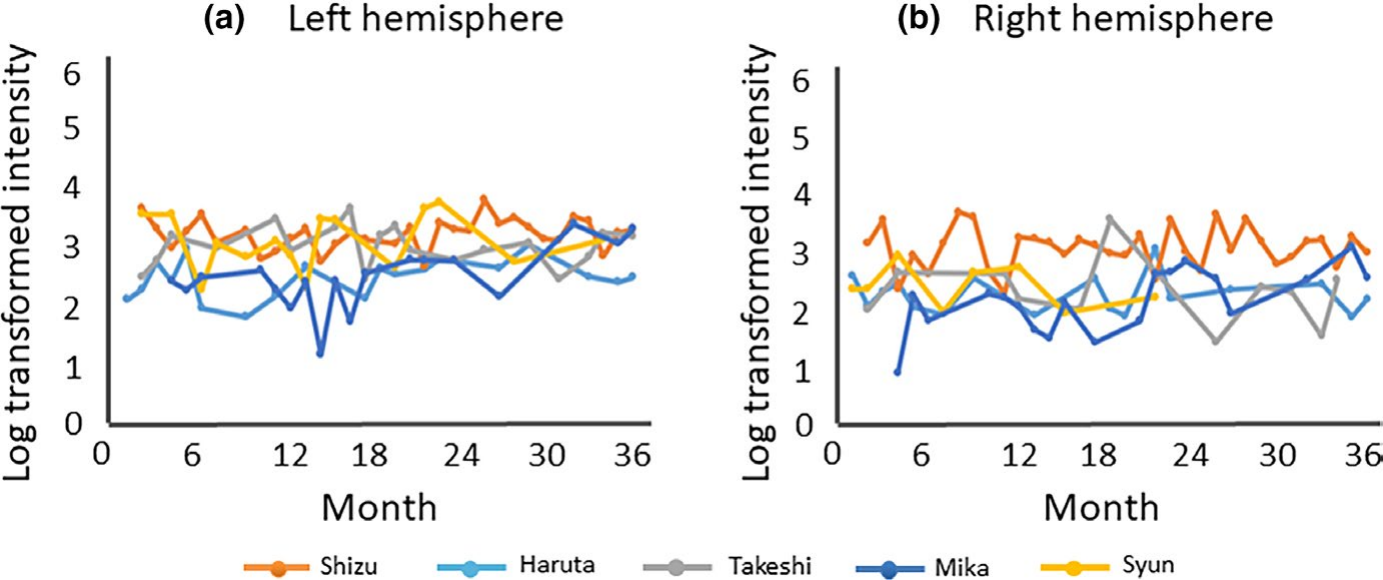
Developmental trajectory of P1m intensity. Left hemisphere (a) and right hemisphere (b). This figure shows the developmental trajectories for all children

The intensity (mean ± *SD*) of P1m for each month is shown in Figure 4. The Jonckheere–Terpstra test failed to demonstrate significant differences with age in P1m intensity in either hemisphere.

**FIGURE 5:**
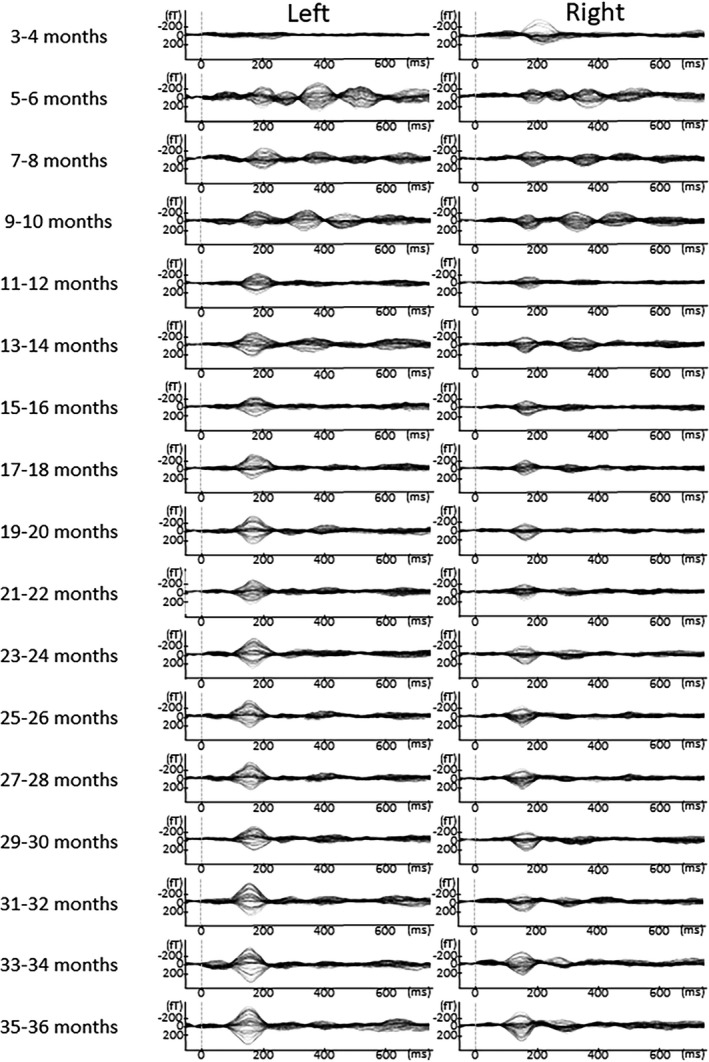
AEF waveforms from each subject at all ages in months. In each hemisphere, 67 sensors were used to record the AEF waveforms. To avoid providing identifying information in this report, the names given to the children here are Shizu

**FIGURE 6:**
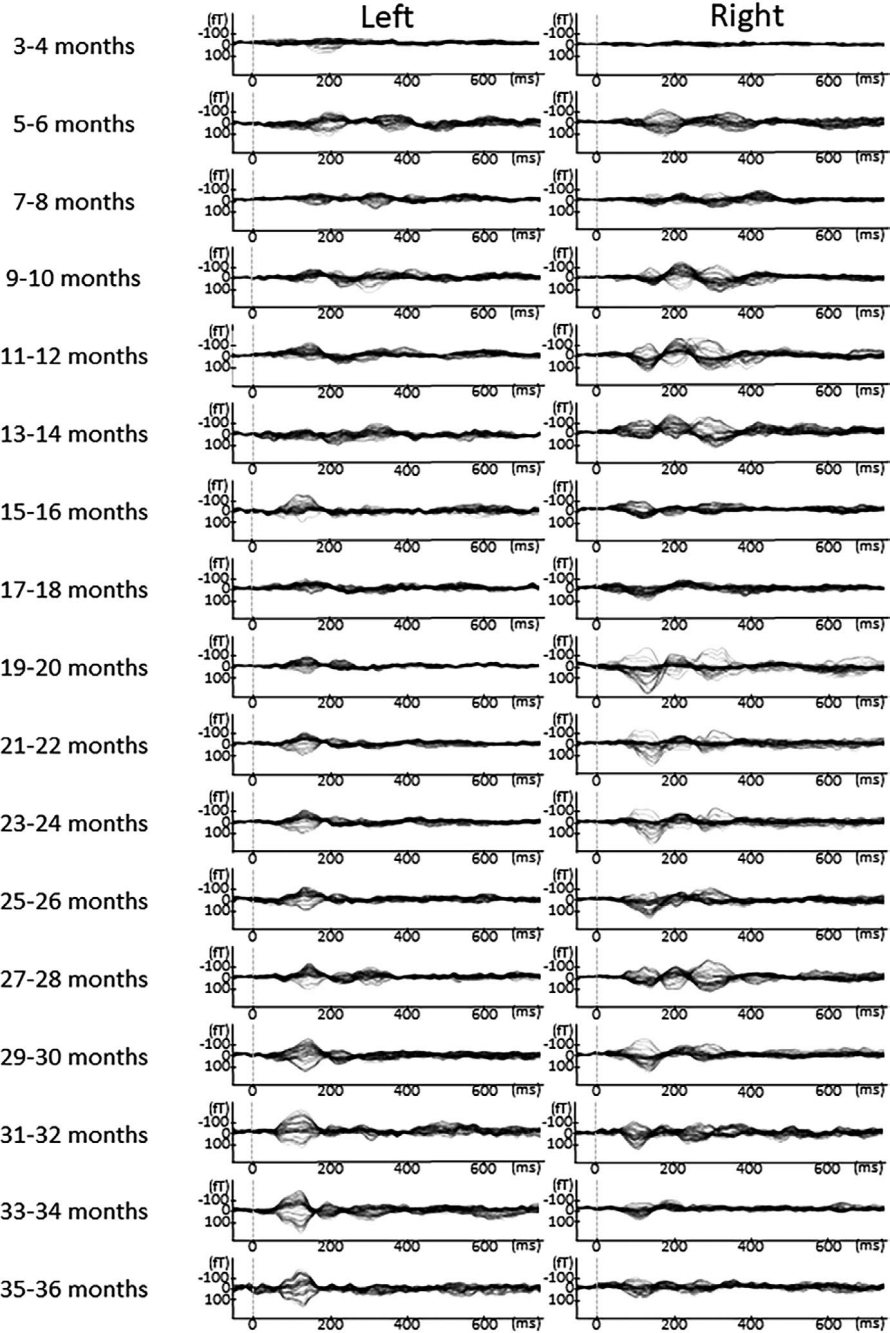
AEF waveforms from each subject at all ages in months. In each hemisphere, 67 sensors were used to record the AEF waveforms. To avoid providing identifying information in this report, the names given to the children here are Haruta

**FIGURE 7:**
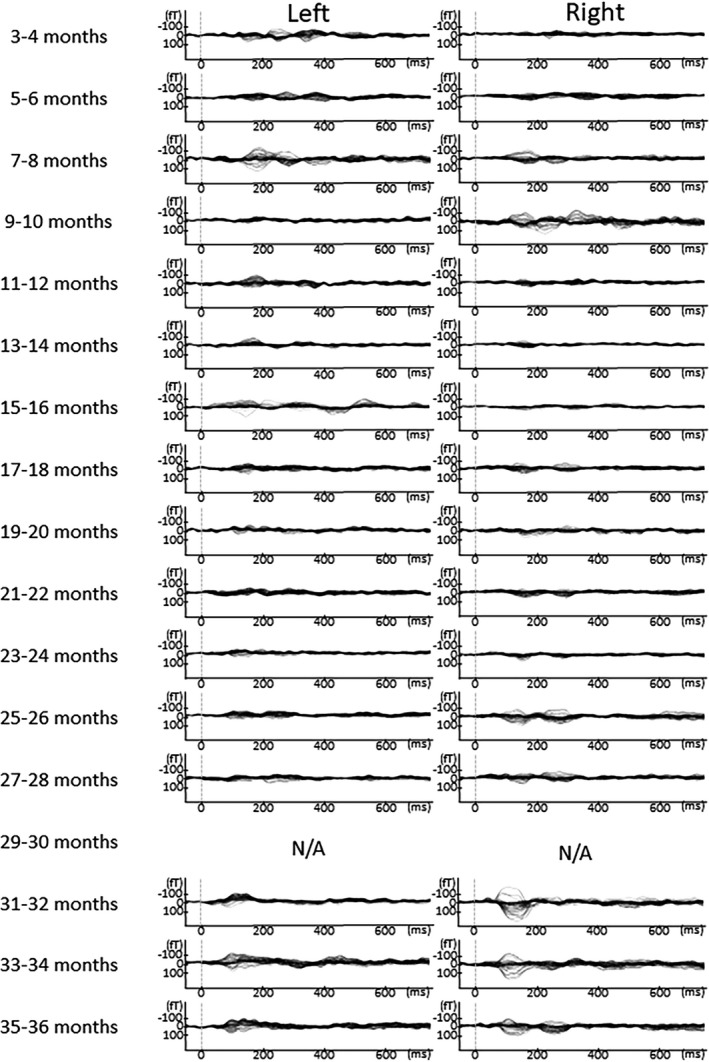
AEF waveforms from each subject at all ages in months. In each hemisphere, 67 sensors were used to record the AEF waveforms. To avoid providing identifying information in this report, the names given to the children here are Takeshi

**FIGURE 8:**
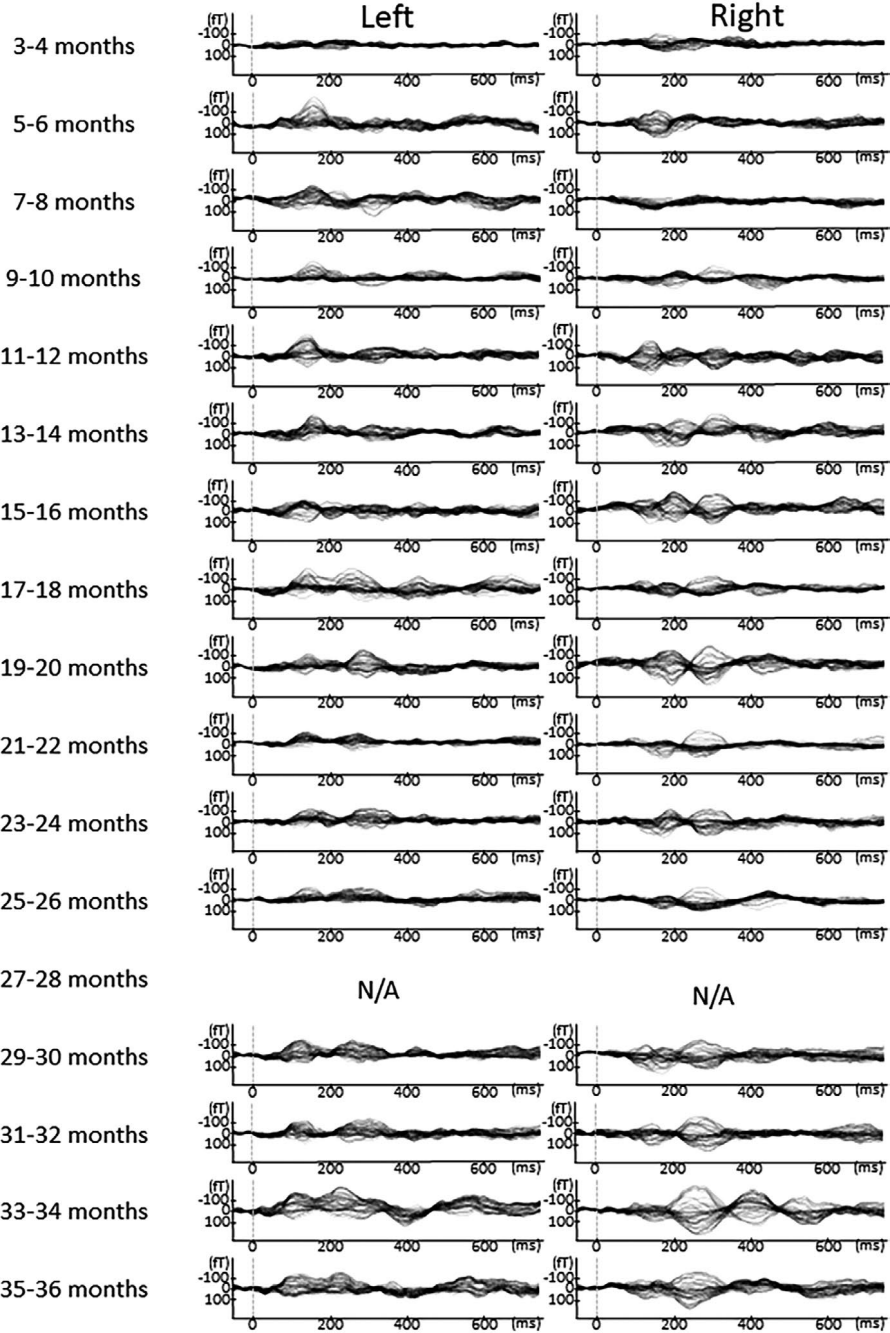
AEF waveforms from each subject at all ages in months. In each hemisphere, 67 sensors were used to record the AEF waveforms. To avoid providing identifying information in this report, the names given to the children here are Mika

**FIGURE 9:**
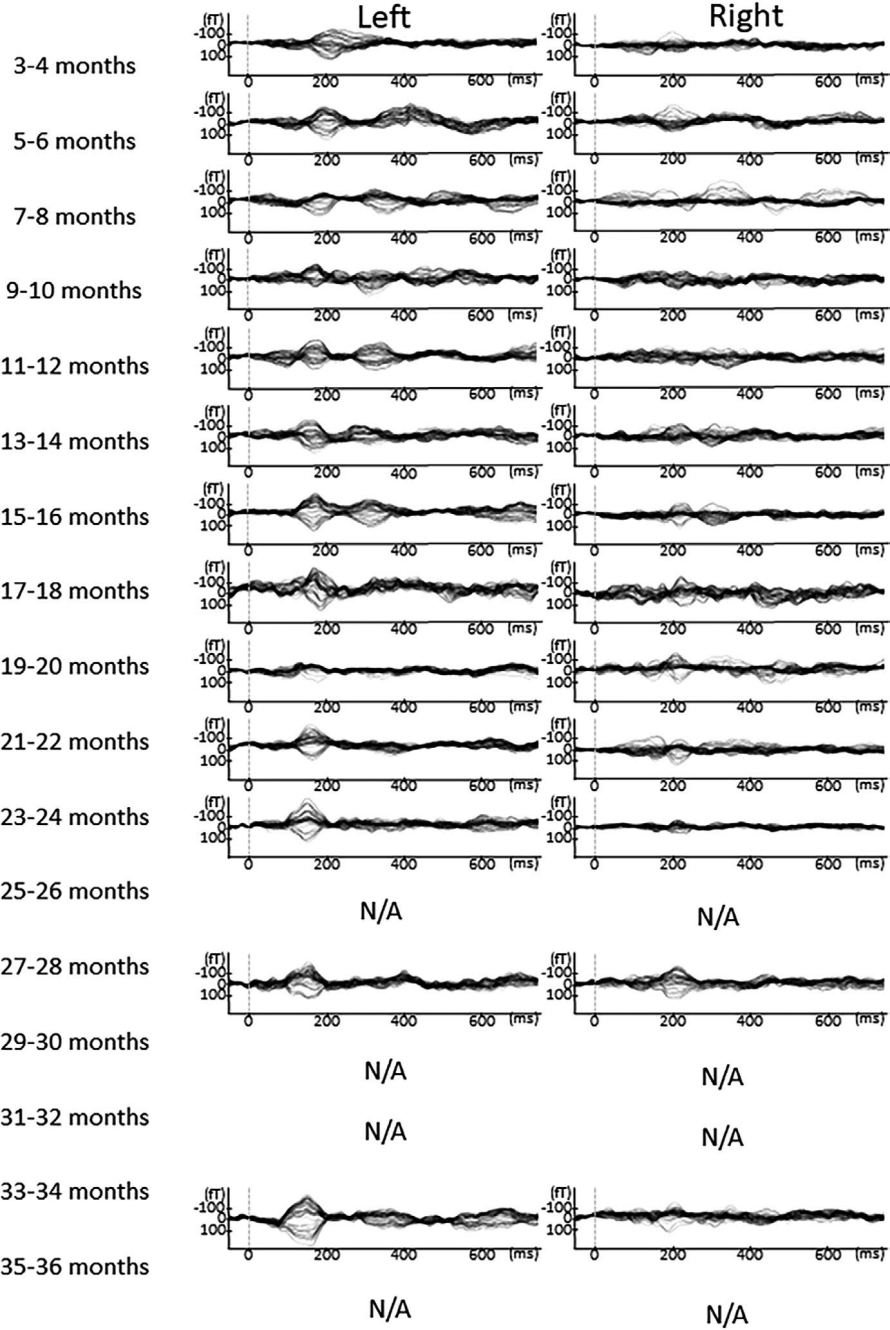
AEF waveforms from each subject at all ages in months. In each hemisphere, 67 sensors were used to record the AEF waveforms. To avoid providing identifying information in this report, the names given to the children here are Syun

In Figures 5, 6, 7, 8, 9, the AEF waveforms for each subject at each different age in months are shown. In each hemisphere, 67 sensors were used to measure the AEF waveforms.

**FIGURE 10:**
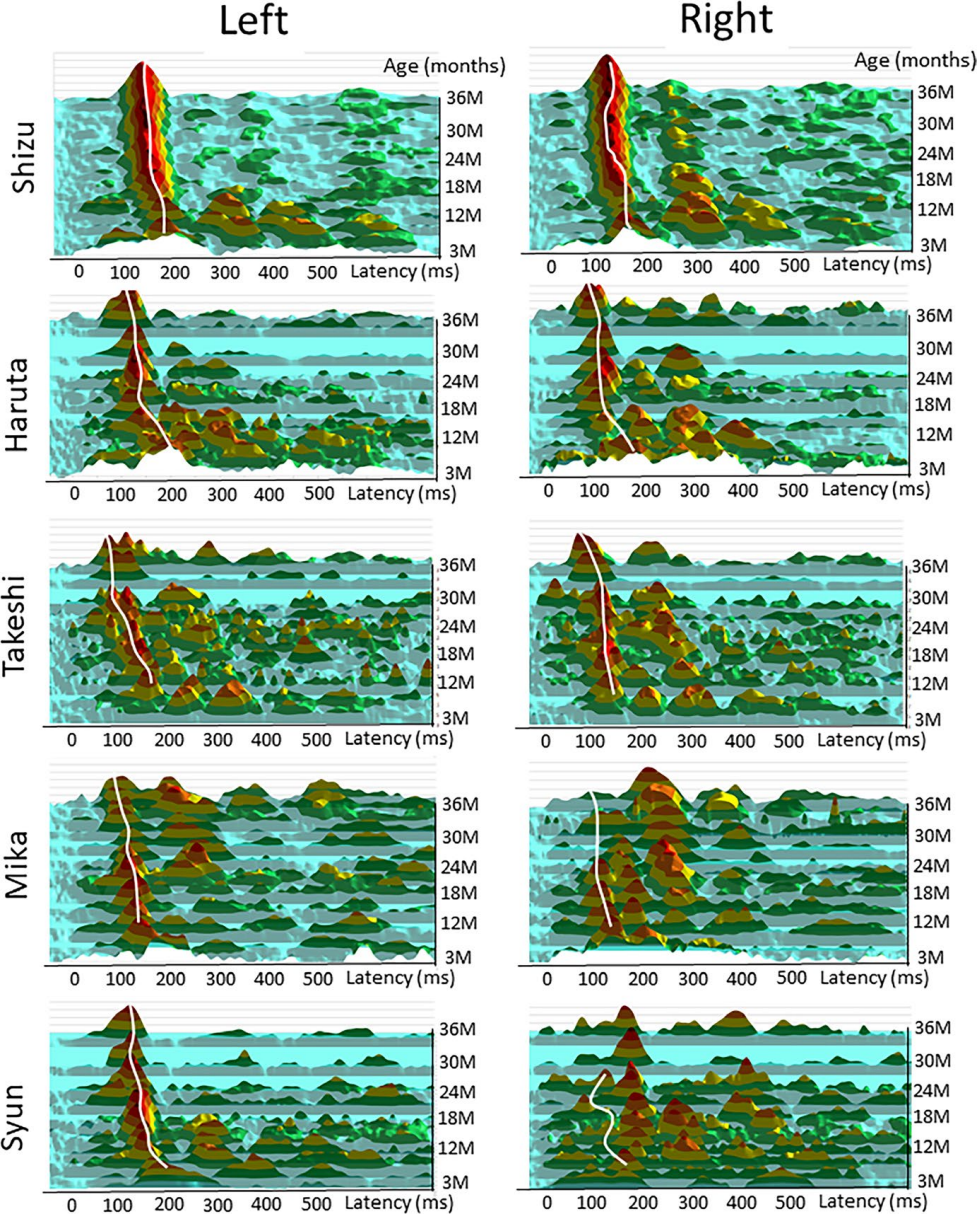
Developmental trajectory of the auditory‐evoked fields from each subject demonstrated by surface plots of the root mean square (RMS) of the magnetic fields for the left and right hemispheres (67 sensors were used for each hemisphere). The RMS values were normalized for each waveform (i.e., the RMS values were divided by the standard deviation of the values in their time windows). Hotter colors (red) indicate greater magnetic field power, and colder colors (blue) indicate lower power. In the left hemisphere, the early prominent positive component (white line) is most prominent in the indicated age range for all subjects

For a comprehensive understanding of the developmental trajectory of the AEFs, surface plots of the root mean square (RMS) of the AEF for the left and right hemisphere are displayed in Figure 10. This figure shows that the early prominent positive component (white line) was most prominent in the indicated age range for all subjects.

## 4 DISCUSSION

### 4.1 The developmental trajectory of the auditory‐evoked early prominent component in subjects under 3 years of age

The data presented here document significant AEF changes associated with age. We investigated the change in the most prominent auditory‐evoked component in infants through MEG measurements approximately every month in 5 typically developing children. The most prominent component that we focused on was estimated in the region corresponding to the left and right temporal lobes by equivalent current dipole estimation using a spherical model. The current source direction of the magnetic field of this component was consistently in the anteroposterior direction (which means electrically positive in the case of EEG central electrodes) from 2 months to 36 months. The latency of the prominent early positive component in infants decreased with age, that is, approximately 200 ms at 2–3 months old and approximately 100 ms at 36 months old. Therefore, as we hypothesized, the early prominent positive component observed in infants aged 0 years was thought to be an immature waveform of the P1m component that we reported in children over the age of 2 years related to language development and/or autism spectrum disorders (Yoshimura et al., 2012, 2013, 2014, 2016). The latency reduction observed here is probably associated with myelination and synaptic efficiency (Eggermont & Salamy, 1988; Ponton et al., 2000). Our results suggest that observing P1m (i.e., the early prominent component) in newborns provides important information for predicting future language acquisition.

### 4.2 The early prominent component detected from fetus to newborn in previous MEG studies

Regarding the cerebral auditory‐evoked responses in subjects from fetuses to newborns, several prior studies seem to have focused on the same “P1m” component in the present study. The components are referred to as the most prominent peak component between 100 and 450 ms from fetus (27 weeks of gestational age) to newborn (Holst et al., 2005). In another newborn (2–12 days of age) study, this component appears on average 280 ms after stimulus onset and was labeled P250m (Huotilainen et al., 2003). Lutter et al. demonstrated this component in children aged 0 years and, according to the latency, they labeled this component P250m (41 weeks of conceptional age) and P150m (51 and 61 weeks of conceptional age) (Lutter et al., 2006). Edgar et al. also demonstrated this component (at a latency of approximately 150 ms) in children aged 6–59 months and labeled it P2m (Edgar et al., 2015). The above components occurred in time windows consistent with that of the “P1m” component investigated in the present study, and despite the different labeling of the components, the direction of the dipole source was the same, that is, in the anteroposterior direction. Therefore, the early prominent components reported in these previous MEG studies on subjects from fetuses to newborns correspond to the “P1m” component in the present study. In addition, although there was no description of dipole direction, two previous MEG studies, one on subjects under 6 months of age (Wakai, Lutter, Chen, & Maier, 2007) and another on subjects more than 3 years of age (Paetau, Ahonen, Salonen, & Sams, 1995), demonstrated that the latency of the early prominent component changes as a function of age, consistent with our results for the “P1m” component.

### 4.3 Various names for early prominent component “P1m” in previous EEG/MEG studies

Inconveniently, the labeling of this early prominent component has not been consistent across different studies. In the present study, we labeled this early prominent component P1m. However, in some previous EEG and MEG studies, this prominent component was labeled with other names when detected in infancy. In previous EEG studies, Wunderlich et al. (2006) reported that a prominent positive peak, P2 (200–250 ms), followed by a prominent negative peak, is typical waveforms in the early months of life (Wunderlich & Cone‐Wesson, 2006). On the other hand, other previous EEG studies labeled this early prominent component P1 (Ceponiene et al., 2003), and consistent with the present study, the latency of P1 has been reported to decrease with age (Cunningham, Nicol, Zecker, & Kraus, 2000; McArthur & Bishop, 2002; Oades, DittmannBalcar, & Zerbin, 1997; Ponton et al., 2000; Sharma et al., 1997). In previous MEG studies, various other names have been given for this early prominent component, for example, “the 250 ms response” in newborns (Huotilainen et al., 2003), “M50” (Oram Cardy et al., 2004; Oram Cardy et al., 2005, 2008; Roberts et al., 2010), “P1m” (Pihko et al., 2007, 2008), or “P50m” (Menning et al., 2005; Onitsuka et al., 2000; Tavabi et al., 2007). We labeled this early most prominent component P1m and previously reported cross‐sectional (Yoshimura et al., 2013, 2016) and longitudinal studies (Yoshimura et al., 2014) on the maturational process of the magnitude of the current source for children aged 2–10 years. This difference in labeling has occurred because most studies are cross‐sectional designs involving narrow age groups, making it difficult to determine which component in one study corresponds to components in the other studies involving other age groups.

### 4.4 Longitudinal changes in early prominent component “P1m” in previous MEG/EEG studies

Only a few studies have reported longitudinal AEF changes with age: Holst from fetus to newborn (Holst et al., 2005), Lutter and Wakai in children aged 0–6 months (Lutter et al., 2006; Wakai et al., 2007), and our previous reported in children aged 3–8 years (Yoshimura et al., 2014). However, there have been no longitudinal reports on the AEF from 0 to 3 years, when brain growth is remarkable. The present MEG study is the first longitudinal study on AEFs from 0‐ to 3‐year‐old children and the first to evaluate the development of the AEF in the left and right hemispheres. On the other hand, there have been a few noteworthy longitudinal AEP studies using EEG. Ohlrich, Barnet, Weiss, and Shanks (1978) investigated changes in auditory‐evoked potential during sleep in very young infants to toddlers (Ohlrich et al., 1978). The results showed that the latency of the P2 component (which corresponds to P1m in the present study), which appeared most prominently in the time window of 100–300 ms, was shortened from 0.5 to 36 months in every sleep stage. Choudhury and Benasich (2011) investigated the cortical auditory‐evoked potentials (AEPs) (i.e., P1, N1, P2, N2) evoked by nonvoice stimuli from 6 to 48 months, and although the type of auditory stimulus was different, consistent with our results, they found that the latency of the AEP component (which corresponds to P1m in the present study) decreased with age in children aged 6 to 48 months (Choudhury & Benasich, 2011). In the present study, at 2–3 months of age, the components that appeared predominantly 150–250 ms after stimulus onset had latencies that decreased as age increased. At 36 months of age, the average of this latency was approximately 100 ms. Notably, the latency and the current direction at 36 months of age in the present study were consistent with the findings for P1m in our previous studies of young children greater than 3 years of age (Yoshimura et al., 2012, 2016).

### 4.5 Longitudinal changes in AEF components other than P1m

Although it was not possible to quantify dipole sources with reliable criteria, we also detected age‐related changes in AEF components other than P1m in the sensor‐level waveforms. In particular, Shizu presented with AEF components that were highly continuous across ages (Figure S1 and S2). Therefore, in the Appendix S1, we added a discussion concerning AEF components other than P1m recorded from Shizu.

## 5 CONCLUSION

This is the first AEF study with a longitudinal design from 0‐ to 3‐year‐old children and the first to evaluate the development of the AEF in the left and right hemispheres. We focused on the early obvious component (P1m) evoked by speech stimuli and conducted a longitudinal study in five typically developing children from 2 to 36 months. As a result, we revealed the relationship between P1m latency and age. These results contribute to the elucidation of the development of brain functions in infants that had not yet been clarified. Both child‐customized MEG and the innovative new technologies in development for the next decade, such as optically pumped magnetometers for MEG (Boto et al., 2018), will provide more crucial information about both typical and atypical brain development in the real world from the newborn stage onward. There are several limitations in this study. First, we could not determine the precise location and current orientation of the dipole source because of a lack of individual MRI structural data. Second, because the five children showed diverse waveforms, the waveform components other than P1m could not be discussed adequately.

## CONFLICT OF INTEREST

All authors declare that there is no conflict of interest.

## AUTHOR CONTRIBUTION

YY was responsible for the original concept and the overall design of the research. CH, HH, and TI collected the MEG data. DNS, TT, and HK contributed to the interpretation of data. YY and MK wrote and revised the manuscript. All authors read and approved the final manuscript.

## Supporting information

Supplementary Material

## Data Availability

The data that support the findings of this study are available on request from the corresponding author. The data are not publicly available due to privacy or ethical restrictions.
