# Supplementary material for: The maturation of the P1m component in response to voice from infancy to 3 years of age: A longitudinal study in young children
Source: Brain Behav. 2020 Jun 23;10(8):e01706. doi: 10.1002/brb3.1706 (PMC7428512; doi:10.1002/brb3.1706)
Supplement: Supplementary file 1 — Supplementary Material [file BRB3-10-e01706-s001.doc]

**Supplementary materials**

**Title: The maturation of the P1m component in response to voice from infancy to 3 years of age: a longitudinal study in young children**

Yuko Yoshimura^1,2^*, Chiaki Hasegawa^2^, Takashi Ikeda^2^, Daisuke N. Saito^2^, Hirotoshi Hiraishi^3^, Tetsuya Takahashi^4^, Hirokazu Kumazaki^2^, and Mitsuru Kikuchi ^2^

1. Institute of Human and Social Sciences, Kanazawa University, Kanazawa, 920-1192, Japan
2. Research Center for Child Mental Development, Kanazawa University, Kanazawa, 920-8640, Japan
3. Institute for Medical Photonics research, Hamamatsu University school of medicine, Hamamatsu, 431-3192, Japan
4. Health Administration Center, University of Fukui, Fukui, 910-1193, Japan

* Corresponding author: Institute of Human and Social Sciences, Kanazawa University, Kakuma-machi, Kanazawa 920-1192, Japan

E-mail address: yukuchen@staff.kanazawa-u.ac.jp

Tel: +81-76-264-5512, Fax: +81-76-264-5510

***Longitudinal changes in AEF components other than P1m.***

Although it was not possible to quantify dipole sources with reliable criteria, we also detected age-related changes in AEF components other than P1m in the sensor-level waveforms. In particular, Shizu presented with AEF components that were highly continuous across ages. Therefore, here we present a discussion concerning AEF components other than P1m recorded from Shizu.

***N0m component***

Regarding the component occurring before P1m, Burnet et al (1975), in an EEG study, reported that a relatively small, sharp, and negative deflection (i.e., N0) occurred with a latency between 13 and 60 ms; N0 components with the shortest latency displayed the weakest relationship to age . In the current study, it is possible that the components that appeared before P1m (a, b, c, and d in Figure S1 and a, b, c, d, and e in Figure S2) were consistent with this N0. However, to verify that the component appearing in the early time window in our study was N0, it is necessary to examine this component in a larger sample size.

***N1m component***

Immediately following P1m, our representative data showed components in which the direction of the current of the magnetic source field was opposite to that of P1m. This component appeared until approximately 9 months of age in the left hemisphere and appeared from several to 13 months and after 27 months in the right hemisphere. Although some EEG studies showed that the P1 component is robustly present even at 5 years of age , the N1b peak (that which follows P1) is not consistently present until 9 years of age and older. On the other hand, in a previous MEG study, Edgar et al. (2015) reported the negative components occurring after P2m (i.e., the component we labelled P1m) as N2m at 6–60 months of age. In this N2m, the mean latency was 230 ms in the left hemisphere and 242 ms in the right hemisphere . This N2m component in Edgar et al’s study may correspond to the negative component we call interim N1 (i.e., Figure S1, i, j, k, and l; Figure S2, k, l, m and n). Moreover, Stephen et al (2017) showed that AEFs typically contained 3 peaks in children aged 6–68 months. Of the three peaks, the early peak (what they call Peak 1) was oriented in the opposite direction to our “P1m”; we consider that early peak to be consistent with the N2m of Edgar et al. (2015) and our “N1m”. Stephen et al. (2017) suggested that a sufficient ISI (2 s) was required for N1m to be detected. Edgar et al. (2015) also mentioned that if the ISI is sufficiently long (1 sec or longer), N1m may be detected even if the child is under 5 years of age. In this study, we used a short ISI (ISI < 1 s). Therefore, we did not identify a reliable N1m component in our data.

***P2m component***

As shown in Figure S1 (m and p) and Figure S2 (o–r), interim P2m components are present, which could be detected more constantly in the right hemisphere. However, we could not find corresponding components in previous studies, which primarily stimulated subjects with tones . Further studies with voice stimuli in subjects from newborn to young children are necessary to define this component.

***N2m component***

As shown in Figure S1 (q and r) and Figure S2 (s–y), our interim N2m component, which could be detected before 14 months in the left hemisphere and consistently in the right hemisphere, seems to be the N2 component reported by Wunderlich et al. (2006) . N2 is a negative component observed in the time window of 300-550 ms after stimulus presentation. In our results, especially in the left hemisphere, we only detected components that seemed to correspond to N2m up to 14 months. We cannot conclude whether the current direction of the magnetic field source changed due to the morphological change in the cerebral sulcus or to physiological changes during maturation.

***P3m component***

In studies on auditory-evoked responses in infants, little has been reported on components appearing in later time windows. Regarding components further in time than N2m, Lutter et al. (2006) investigated longitudinal brain responses to tones in newborns (36-42 weeks of conceptional age) up to 6 months of age. As a result, they reported that downward components appear as N750m, whose latency decreased with age. Intriguingly, at 50-52 weeks of conceptional age (corresponding to 2–3 months of age), they also reported a mean latency of 558 ms. These seem to correspond to our results (latency, 550–620) shown in Figure S1 (v and w) and Figure S2 (D and E). According to a previous study in subjects aged 0.5–36 months , P3 was the major positive wave following N2 and could have a P3A form, a prominent positive deflection followed by a negative trough, or a P3B form, a broad positive wave lacking a well-defined peak. A P3A wave could be followed by a P3B wave of greater positivity, and the mean P3A latency was 660 ms at 0.5 months of age . In the present study, as shown in Figure S1 (v) and Figure S2 (D), an interim P3m component (latencies, 602 ms in the left and 618 ms in the right hemisphere at 5 months of age) was detected after the interim N2m component. This interim P3m may correspond to the P3 component (P3A, P3B) observed in a previous EEG study .

Supplementary figures


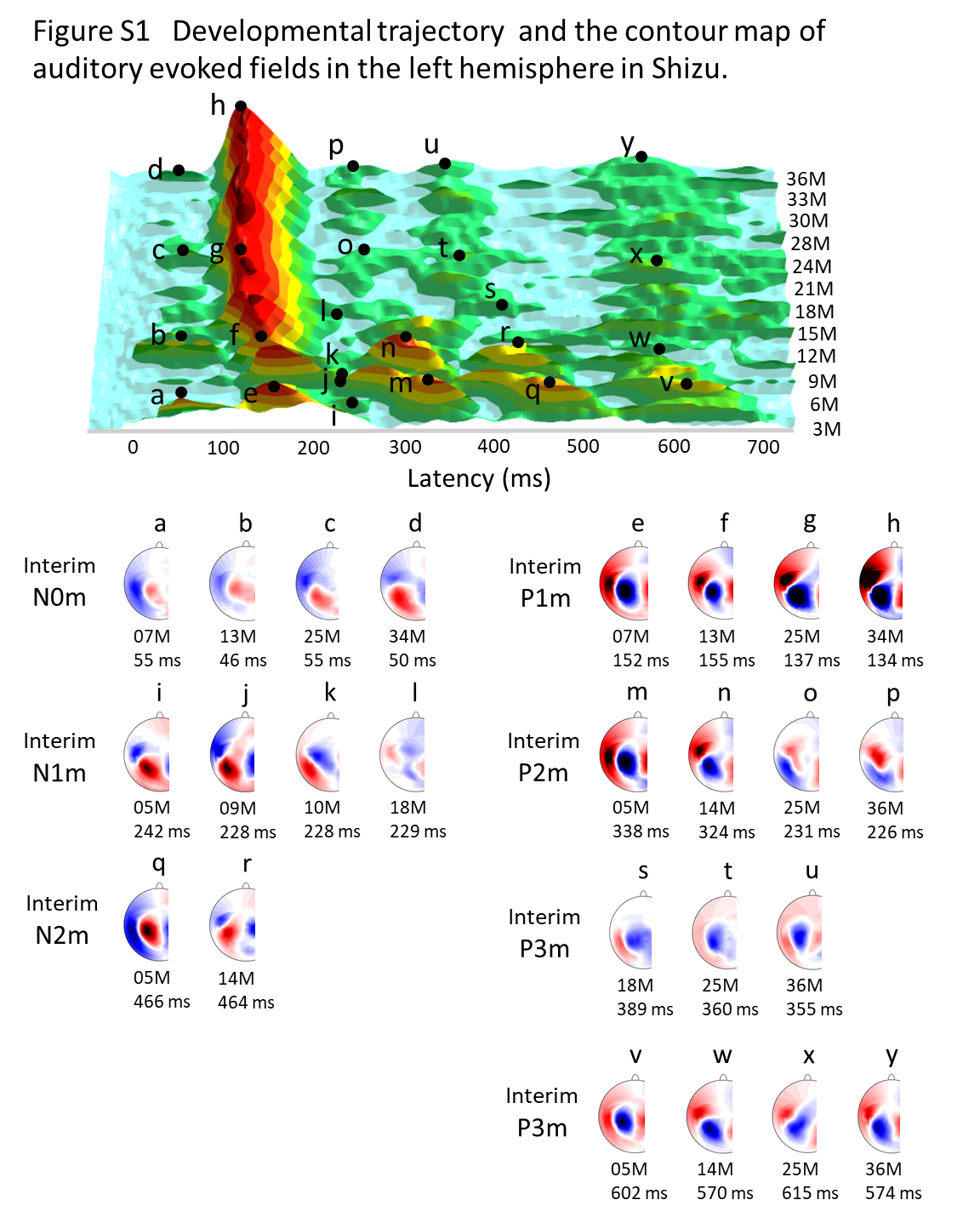


Figure S1 (Upper figure) Developmental trajectory of the AEF from one subject demonstrated with a surface plot of the root mean square of the magnetic fields from the left hemisphere. Hotter colours (red) indicate greater magnetic field power, and colder colours (blue) indicate lower power. The P1m component (black dots: e-h) is most prominent in this age range, and the P1m latency is nearly constant as a function of age. (Lower figures) The contour maps for some detectable components of the auditory-evoked fields from sensors placed above the left hemisphere.


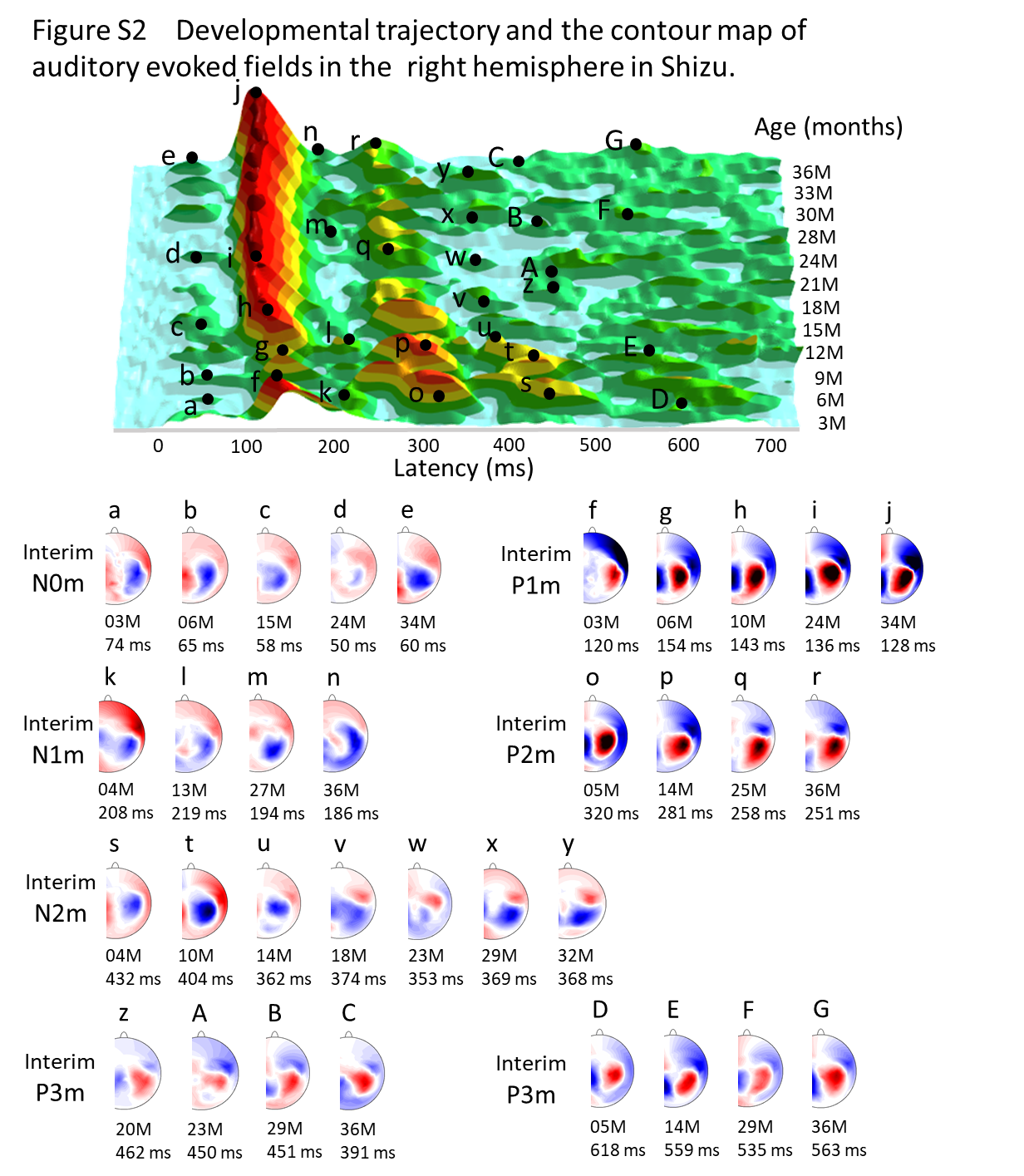


Figure S2 (Upper figure) Developmental trajectory of the auditory evoked field from one subject demonstrated with a surface plot of the root mean square of the magnetic fields from the right hemisphere. Hotter colours (red) indicate greater magnetic field power, and colder colours (blue) indicate lower power. The P1m component (black dots: e-h) is most prominent in this age range, and the P1m latency is nearly constant as a function of age. (Lower figures) The contour maps for some detectable components of the auditory-evoked fields from sensors placed above the right hemisphere.

Supplemental table 1. The number of cases where the ECD could be modelled after meeting the criteria in the main text at each age in months.

|  | Number of cases where the ECD could be modelled after meeting the criteria/Number of MEG measurements | |
| --- | --- | --- |
|  | Left hemisphere | Right hemisphere |
| 2 months | 1/3 | 2/3 |
| 3 months | 4/4 | 4/4 |
| 4 months | 3/4 | 2/4 |
| 5 months | 5/5 | 5/5 |
| 6 months | 3/3 | 3/3 |
| 7 months | 4/4 | 2/4 |
| 8 months | 4/5 | 3/5 |
| 9 months | 0/3 | 1/3 |
| 10 months | 3/4 | 3/4 |
| 11 months | 2/3 | 2/3 |
| 12 months | 5/5 | 3/5 |
| 13 months | 4/4 | 4/4 |
| 14 months | 4/4 | 3/4 |
| 15 months | 3/3 | 2/3 |
| 16 months | 4/4 | 3/4 |
| 17 months | 3/4 | 2/4 |
| 18 months | 4/4 | 3/4 |
| 19 months | 3/3 | 3/3 |
| 20 months | 4/5 | 2/5 |
| 21 months | 3/3 | 2/3 |
| 22 months | 4/4 | 4/4 |
| 23 months | 3/4 | 3/4 |
| 24 months | 3/3 | 2/3 |
| 25 months | 1/1 | 1/1 |
| 26 months | 2/3 | 3/3 |
| 27 months | 3/3 | 3/3 |
| 28 months | 2/2 | 1/2 |
| 29 months | 3/3 | 2/3 |
| 30 months | 1/1 | 1/1 |
| 31 months | 2/2 | 2/2 |
| 32 months | 2/3 | 2/3 |
| 33 months | 3/3 | 3/3 |
| 34 months | 3/3 | 2/3 |
| 35 months | 3/3 | 3/3 |
| 36 months | 4/4 | 3/4 |

**References**

Barnet, A. B. (1975). Auditory evoked potentials during sleep in normal children from ten days to three years of age. *Electroencephalography and clinical neurophysiology, 39*(1), 29-41.

Edgar, J. C., Lanza, M. R., Daina, A. B., Monroe, J. F., Khan, S. Y., Blaskey, L., . . . Roberts, T. P. (2014). Missing and delayed auditory responses in young and older children with autism spectrum disorders. *Frontiers in human neuroscience, 8*, 417. doi:10.3389/fnhum.2014.00417

Lippe, S., Martinez-Montes, E., Arcand, C., & Lassonde, M. (2009). Electrophysiological study of auditory development. *Neuroscience, 164*(3), 1108-1118. doi:10.1016/j.neuroscience.2009.07.066

Lutter, W. J., Maier, M., & Wakai, R. T. (2006). Development of MEG sleep patterns and magnetic auditory evoked responses during early infancy. *Clinical neurophysiology : official journal of the International Federation of Clinical Neurophysiology, 117*(3), 522-530. doi:10.1016/j.clinph.2005.11.003

Ohlrich, E. S., Barnet, A. B., Weiss, I. P., & Shanks, B. L. (1978). Auditory evoked potential development in early childhood: a longitudinal study. *Electroencephalography and clinical neurophysiology, 44*(4), 411-423.

Ponton, C., Eggermont, J. J., Khosla, D., Kwong, B., & Don, M. (2002). Maturation of human central auditory system activity: separating auditory evoked potentials by dipole source modeling. *Clinical neurophysiology : official journal of the International Federation of Clinical Neurophysiology, 113*(3), 407-420.

Wunderlich, J. L., Cone-Wesson, B. K., & Shepherd, R. (2006). Maturation of the cortical auditory evoked potential in infants and young children. *Hearing Research, 212*(1-2), 185-202. doi:10.1016/j.heares.2005.11.010
